# Implications of spatial and seasonal air pollution patterns, socioeconomic disparities, and 15-minute communities for achieving WHO air quality guidelines

**DOI:** 10.1038/s41598-025-98123-8

**Published:** 2025-04-21

**Authors:** Bram Vandeninden, Brecht Devleesschauwer, Martina Otavova, Charlotte Vanpoucke, Hans Hooyberghs, Christel Faes, Catherine Bouland, Eva M. De Clercq

**Affiliations:** 1 School of public health, Université Libre de Bruxelles, Brussels, Belgium; 2 Department of Epidemiology and Public Health, Sciensano, Brussels, Belgium; 3 Department of Chemical and Fysical health risks, Sciensano, Brussels, Belgium; 4 Center for Demographic Research, UCLouvain, Louvain-La-Neuve, Belgium; 5 Interuniversity Institute for Biostatistics and statistical Bioinformatics (I-BioStat), Data Science, Diepenbeek, Hasselt, Belgium; 6 Department of Translational Physiology, Infectiology and Public Health, Ghent University, Merelbeke, Belgium; 7 Belgian Interregional Environment Agency, Brussels, Belgium; 8 VITO, Flemish Institute for Technological Research, Mol, Belgium

**Keywords:** Environmental impact, Risk factors

## Abstract

Achieving WHO air pollution guidelines is critical to reduce the health burden of air pollution, which disproportionately affects socioeconomically disadvantaged populations and varies by sector, spatial distribution, and seasonal trends. This study explores the implications of sectorial and spatial-seasonal air pollution patterns, socio-economic disparities, and 15-minute communities to achieve (2021) WHO air quality guidelines for PM_2.5_ and NO_2_. The study analyses spatial-temporal patterns of air pollution in Belgium. Seasonal air pollution exposure is assessed through summer-to-winter ratios, stratified by land cover, urbanisation, and proximity to roads, and linked to socio-economic disparities using LOESS regression. A case study evaluates the mitigation potential of 15-minute communities for traffic-related air pollution, leveraging the Mobiscore tool to explore the relationship between accessibility and car ownership, a proxy for traffic-related emissions. NO_2_ and PM_2.5_ show marked seasonal and spatial variations, with higher concentration ratios in summer near busy roads and urban centres, especially for NO_2_. In general the NO_2_ spatial-seasonal pattern is more heterogenous compared to the PM_2.5_ pattern. Winter pollution exposure significantly hampers meeting WHO health targets, although summer levels of NO_2_ remain high around major traffic routes. The observed disparities in exposure to NO₂ highlight significant socio-economic inequalities, with the most deprived populations disproportionately burdened by traffic-related air pollution. The results from our case-study to mitigate traffic-related air pollution demonstrate that, up to a Mobiscore of 8.0, car ownership remains constant with increasing availability of services and public transport. From a turning point Mobiscore of 8.0, car ownerships starts to drop significantly, indicating that improving Mobiscores to very high scores ( > = 8.0) may lead to reduced car ownership and lower NO_2_ and PM_2.5_ emissions and exposure. Our study highlights important spatial-seasonal patterns in air pollution and their health implications, emphasizing the need for season-specific and structural traffic interventions to meet WHO guidelines for PM_2.5_ and NO_2_ exposure. A case study on mitigating traffic-related air pollution identifies a threshold where sufficient public transport and service accessibility lead to a reduction in car ownership. Addressing socio-economic disparities is crucial, as these areas often face greater challenges in meeting WHO air pollution guidelines, particularly for NO₂.

## Introduction

Long-term exposure to Nitrogen Dioxide (NO_2_) and Particulate Matter < 2.5 μm (PM_2.5_) considerably contributes to disease, hospitalizations and mortality, by increasing the risk of cardiovascular disease, asthma, brain damage, Alzheimer disease and cancer^1–4^. In addition, long-term and short-term exposure to these air pollutants can increase both the severity and incidence of respiratory infections including influenza and COVID-19^5,6^. Relations between short-term fluctuations in air pollutants and elevated blood pressure, cardiovascular events, and infectious diseases are well-documented in existing literature^7,8^.

Seasonal patterns exist in cardiovascular events such as myocardial infarctions and infectious diseases like influenza, COVID-19, and bacterial pneumonia, with a higher occurrence during winter^9–11^.

Environmental factors, including non-optimal temperatures (cold and extreme heat) and air pollution, can contribute to those seasonal patterns of diseases. Air pollution levels display dynamic temporal variations, influenced by factors such as traffic patterns, meteorological conditions, and atmospheric chemistry. Factors playing a role here are amongst others emissions (e.g., wood burning for residential heating peaks in winter but is almost absent in summer) and changes in the height of the atmospheric mixing layer, which affects pollution dispersion^12^. Processes can be often complex. For example, for PM_2.5_, Ammonia (NH₃), black carbon (BC), and organic carbon (OC) are consistently dominant precursors, with their seasonal variations closely linked to agricultural activities and residential energy use, particularly during colder months. Agriculture (both crops and livestock) and residential heating emerge as leading sources of PM_2.5_emissions, especially during winter due to increased heating demands. Meanwhile, road transport and industrial activities remain significant contributors throughout the year^13^.

Seasonal patterns of air pollution are particularly relevant as they influence exposure levels and population vulnerability in ways that differ across regions and timeframes. Existing research on seasonal patterns provides valuable insight but highlights considerable variation depending on geographic and climatic contexts^14–17^. Furthermore, to the best of our knowledge, spatial components of these seasonal variations—how they manifest and interact across different areas—are often not considered. Addressing these gaps is critical to advancing our understanding of air pollution dynamics and tailoring effective mitigation strategies, for example to be able to reach WHO guideline values of air pollution at all locations.

In addition, source allocation of air pollutants to sectors is often complex, while it has important implications for mitigating air pollution levels to achieve the WHO guidelines of 5 and 10 µg/m³ for PM_2.5_ and NO_2_. In depth-analysis of the spatial-seasonal patterns can contribute to understanding some of the patterns of contribution of different factors to air pollution in different seasons. The **first main objective** of this research is to investigate the spatial-seasonal variations in Belgium and stratifying seasonal variations in air pollution concentrations per land cover class such as industry, agriculture, proximity of roads, residential areas and degrees of urbanisation, potentially deducting information on (season-specific) source allocation of air pollutants.

Environmental factors, including air pollution, are frequently linked to socio-economic variables such as education level and income. These associations can be mixed and multifaceted^18,19^. The **second main objective** of this research is to examine socio-economic disparities. Specifically, we focus on the relationship between annual and spatio-seasonal air pollution levels and socio-economic inequalities, analysed separately for urban and rural areas.

The **third main objective** in this research is a case-study to investigate whether a mitigation measure in the mobility and urban planning domain, namely increasing the amount of services and public transport in neighbourhoods, closely linked to the concept of 15-minutes that is gaining attention, is associated with lower car ownership per household and therefore lower emissions, potentially being able to contribute in order to reach the WHO guidelines of 5 µg/m³ PM_2.5_ and 10 µg/m³ NO_2_long-term exposure to air pollution^20^.

## Data and methodology

### Study area

Belgium is a country in Europe, within the European Union (EU). It has a moderate climate with mild maritime weather, dominated by southwest winds, abundant cloud cover, and frequent precipitation. As of 2023, Belgium has 11.6 million inhabitants, of which 21% live in the five largest cities: Brussels (1.23 million), Antwerp (0.53 million), Ghent (0.26 million), Charleroi (0.21 million), and Liège (0.20 million).

**Fig. 1:**
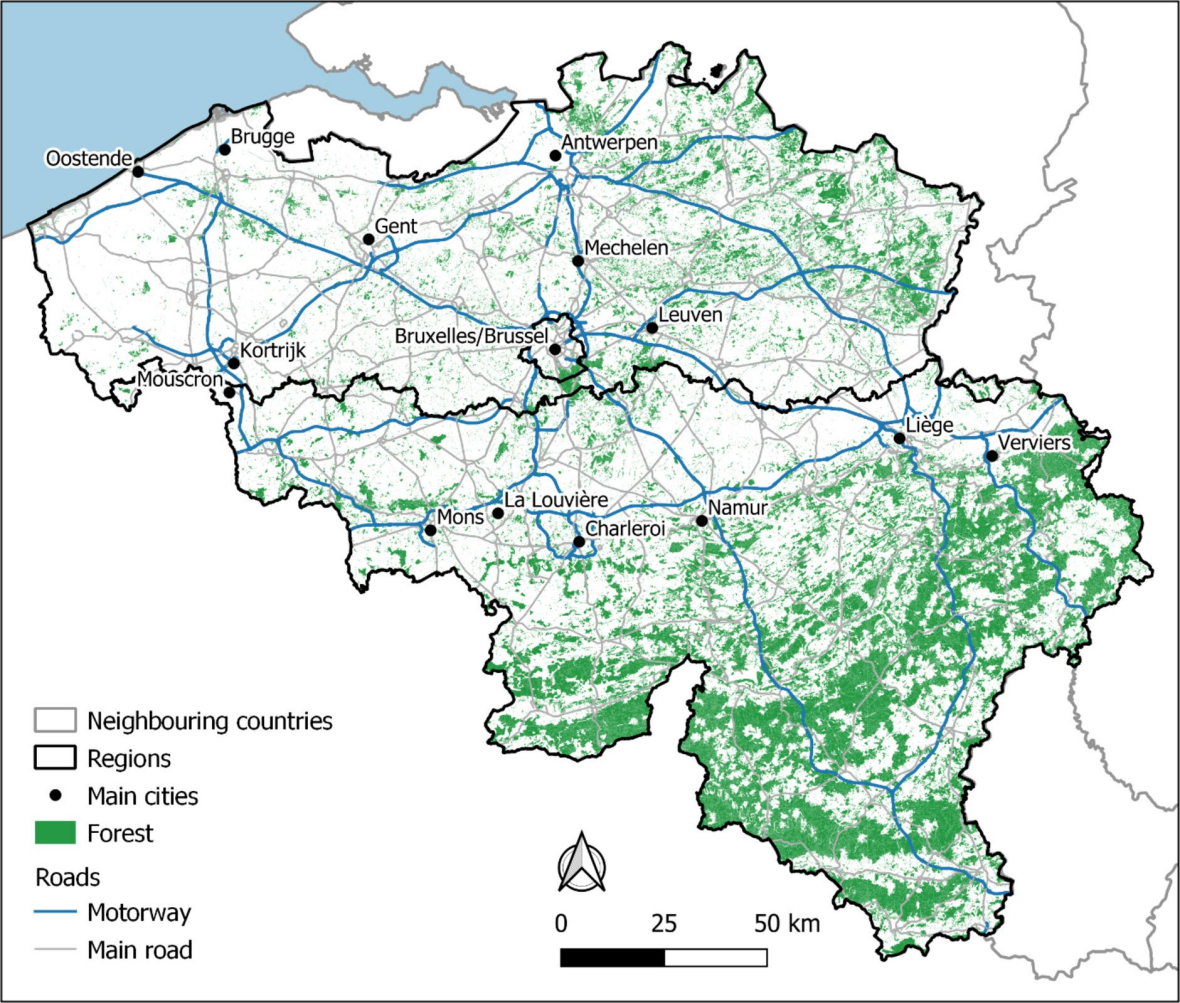
Map of the study area. Made with top10 vector, https://www.geo.be/catalog/details/e0fdc885-8851-482e-80c9-6a0ba3709761?l=en.

The map below (Fig. 1) displays Belgium and its surrounding countries, including the North Sea to the northwest. The map contains motorways, major roads (line elements), major cities (labelled on the map), large forested areas (dark green), and the country borders of Belgium (thick bold black line).

### Data

Seasonal air pollution data for nitrogen dioxide (NO₂) and particulate matter (PM_2.5_) were obtained from IRCEL-CELINE (https://irceline.be) for the years 2016 and 2019. The seasons are defined according to meteorological classifications: winter (December, January, February), spring (March, April, May), summer (June, July, August), and autumn (September, October, November). Seasonal maps are based on monthly generated maps from the RIO-IFDM model chain^21–23^.

The study used data from 2016 to 2019, with monthly maps provided by IRCEL, the Belgian Interregional Environment Agency, which were derived from hourly model outputs. In our analysis, we spatially aggregated the 10 m pollution levels to air pollution levels at the level of the statistical sector.

Exposure-Response Functions (ERFs) for the relation between air pollutants such as NO_2_ and PM_2.5_ and all-cause mortality were derived from existing meta-analyses, as further explained in the methodology.

In addition, several open-data datasets are used during the research, including CORINE 2018 Land Cover Data (Copernicus, https://land.copernicus.eu/en/products/corine-land-cover/clc2018). We used the CORINE Land Cover 2018 dataset, which provides a pan-European land cover inventory with 44 thematic classes for the 2018 reference year, updated every six years. For Belgium, we utilised the 100 m raster format, which features a minimum mapping unit of 25 hectares for areal phenomena and a minimum mapping width of 100 m for linear phenomena.

Further, number of cars owned per household^24^, Mobiscore^25^and Degree of Urbanisation^26^are used. The number of cars owned per household is available as a dataset at the statistical sector level, and in our research the metric is used as number of cars owned per household at the statistical sector level. The degree of urbanisation (DEGURBA) is a classification system that categorizes areas along an urban-rural continuum into three distinct classes—cities, towns and suburbs, and rural areas—based on population size and density thresholds. DEGURBA is available for every commune in Belgium. Communes consist out of multiple statistical sector, implying that all statistical sectors within one commune have the same DEGURBA score. The Mobiscore is a tool developed that provides an overview of how well essential services such as education, groceries, doctors, recreational and sport services and public transport such as railway stations and bus stops are accessible within a reasonable distance from place of residence^25^. The Mobiscore is a composite index taking into account the categories (A) public transport, (B) education, (C) shops & services, (D) recreation, sport & culture and (E) Health services. Based on an address, the proximity of a residence to amenities such as shops, schools, train stations, and bus or tram stops is assessed, considering average travel patterns in Flanders. Factors include the frequency of visits to specific amenities, the distance to nearby amenities, the mode of transport used, and its environmental impact^25^. The Mobiscore is available as a grid raster. We aggregated the Mobiscore to one value per statistical sector for analytical purpose.

To analyse socio-economic disparities, we use the Belgian Index of Multiple Deprivation (BIMD). The BIMD2011 is a comprehensive tool designed to assess multidimensional deprivation across Belgium, tailored to specific time periods and spatial contexts. It provides measurements at the level of statistical sectors, the smallest geographic units in Belgium. This index evaluates deprivation across six key domains: income, education, employment, housing, health, and crime. These domains are aggregated to produce a single composite score, representing the overall level of deprivation for each area^27^.

## Methodology

### Seasonal and spatio-seasonal air pollution patterns

The air pollution data used in this study were derived from an integrated model chain known as IFDM^23^. The RIO land-use regression model^21^calculates the regional contribution to air pollutant concentrations at a resolution of 4 × 4 km² using hourly measurements from official monitoring stations. The IFDM Gaussian dispersion model^22^estimates local increments resulting from road transport, shipping emissions, and point sources. Emissions from point sources (e.g., industrial sources) and line sources (road traffic, shipping) are incorporated into the IFDM, while other potential sources such as households (e.g., wood-burning stoves) and agriculture are considered when determining RIO background concentrations. As a receptor model, it computes pollutant concentrations at specific receptor sites based on the provided emission inputs. The study analysed data from 2016 to 2019, using monthly maps derived from model outputs at an hourly temporal resolution, which were subsequently aggregated into seasonal maps. For this study, we spatially aggregated the high-resolution (10 m) maps produced by the model chain to the level of the statistical sector, which is the smallest administrative unit in Belgium. This aggregation ensured compatibility with our study design while maintaining spatial detail to capture variations in pollution exposure. The model has been thoroughly validated, demonstrating strong performance in accurately representing air pollution levels^23^. This approach offers advantages over alternatives such as coarser-resolution chemical-transport models or the sparse spatial coverage of air quality monitoring stations.

Air pollution concentrations are spatially aggregated to the level of the statistical sector, the smallest administrative unit in Belgium, using the ‘Zonal Statistics as Table’ tool in ArcGIS (https://pro.arcgis.com/en/pro-app/latest/tool-reference/spatial-analyst/zonal-statistics-as-table.htm). This approach combines fine-scale 10 × 10 m air pollution data into the broader statistical sector level, which represents relatively homogeneous areas with an average population of approximately 500 people.

In this study, we present the seasonal variations in air pollution patterns through a map that demonstrates the ratio between summer and winter air pollution concentrations. This is analysed at the level of the statistical sector, allowing for a clear visualisation of the spatial distribution of this ratio within Belgium. Additionally, a graph is included to display the ratios for summer, spring, and autumn, using winter as the reference season for air pollutants considered. Maps are produced with ArcGIS version 10.8 and graphs with the statistical software tool R^28,29^. The summer-to-winter concentration ratio is obtained by dividing the mean summer concentration by the mean winter concentration.

In addition, a more in-depth analysis of spatial-seasonal patterns was performed by (a) calculating the summer/winter ratio of NO_2_ and PM_2.5_ stratified per CORINE land cover class including 95% CI (formula 1). The CORINE Land Cover (CLC) 2018 dataset is considered representative for this study, which covers the year 2019. This is justified by the relatively static nature of land cover, as substantial changes in land use typically occur over extended periods rather than within a few years (b) calculating the summer/winter ratio of NO_2_ and PM_2.5_ divided in three categories reflecting degree of urbanisation (formula 2), (c) plotting a linear and localised-linear regressions of the relation between the summer/winter ratio of NO_2_ and PM_2.5_and the distance to motorways, primary and secondary roads. For this analysis, a path_distance is calculated with a built-in ArcGIS tool and a 95% CI is plotted too. The localised-linear regression is made with LOESS in ggplot in R^30,31^.

$$\begin{aligned} & \:\left(a,\:formula\:1\right)\:ratio=\frac{summer\:concentration\:(LC\:class=k)}{winter\:concentration\:(LC\:Class=k)},\\ &\:\:k\in\:\left\{continuous\:urban\:fabric,\:discontinuous\:urban\:fabric,\:industrial\:or\:commercial\:units,\:road\:and\:rai\dots\:,\:\dots\:.\right\}\end{aligned}$$

$$\:\left(b,\:formula\:2\right)\:\:\:ratio=\frac{Summer\:Concentration\:\left(DEGURBA=k\right)}{Winter\:Concentration\:(DEGURBA=k)},\:k\in\:\left\{\text{1,2},3\right\}with\:k=1:Urban;k=2:Town,\:k=3:rural$$

In our analysis, we used the R statistical software^29^along with the ‘ggplot2’ and ‘tidyverse’ packages for data visualization and analysis. The ‘ggplot2’ package^31^is designed to help create clear and visually appealing graphics. The ‘tidyverse’ includes a set of user-friendly tools that simplify tasks such as organizing, transforming, and exploring data^32^.

### Annual population preventable fraction for PM_2.5_ and NO_2_

In our study we calculated mean air pollution values per statistical sector based on input high-resolution 10 × 10 m seasonal IFDM rasters which we specifically created for this study. The subsequent Health impact assessment is conducted by aggregating the high-resolution concentrations to concentrations at the statistical sector level, the smallest administrative unit in Belgium. The Relative Risk (RR) and Preventable Fraction (PF) were calculated at the level of the statistical sector. It is important to note that both the ERFs used from literature as the used air pollution concentrations deal with residential air pollution exposure.

Based on the RR for adult all-cause mortality reported in the ELAPSE meta-analysis, and the counterfactual scenario of reaching the WHO air pollution guidelines for long-term exposure of 5 µg/m³ PM_2.5_ and 10 µg/m³ NO_2_, we calculate the Preventable fraction (% of mortality that could be prevented if exposed to the counterfactual scenario instead of the current reality). The ELAPSE meta-analysis is a comprehensive meta-analysis compiling the most recent cohort studies. The RR corresponds to 1.045 [95% CI: 1.026–1.065] per 10 µg/m^3^ increase in NO_2_ and 1.118 [95% CI: 1.06–1.179] per 10 µg/m^3^ increase in PM_2.5_^33^

The Preventable Fraction (PF) is calculated as follows:

$$\:PF=1-\frac{1}{RR}\:$$

in which $\:RR$ is the relative risk of the exposure.

The RR increases within the concentration range and is rescaled accordingly to match the air pollution concentrations per statistical sector. The RR is recalculated from the default unit per 10 µg/m³ increase to the relevant exposure unit (expressed in µg/m³), using

$$\:RRexposure=exp\left(\right(ln\left(RR10\right)/10\left)*\right(CON\left)\right)$$

The value of RR10 represents the relative risk associated with a 10 µg/m³ increase in the concentration of an air pollutant (listed in the first paragraph of this section including CI), while CON represents the average concentration per season of a pollutant in the relevant statistical sector. As the reported relative risk (RR10) from literature is typically reported as a point estimate with 95%CI, we do want to take into account the uncertainty of this estimate. We therefore calculate confidence intervals of the RRexposure and of the PF using a Monte Carlo simulation (10000 iterations) by assuming that the RR10 comes from a triangular distribution with most likely value given by the point estimate, and lower and upper values gives by the 95% CI.

To aggregate the PF for each statistical sector to one total number for Belgium, the population-weighted sum is taken, accounting for the number of people living in each statistical sector.

### Analysis of socio-economic disparities

The Belgian Index of Multiple Deprivation (BIMD) is a composite index designed to quantify deprivation across several domains, including education, income, housing, employment, crime, and health^27^. Within the housing domain, for example, the index incorporates indicators such as the proportion of individuals living in substandard dwellings (e.g., smaller than 35 m²), the proportion of tenants, and the proportion of individuals without basic amenities like central heating, insulation, a kitchen, a toilet, a bathroom, or internet access. A complete list of subcriteria used in constructing the BIMD is detailed in the original publication^27^.

The BIMD categorizes the population into 10 deciles, with Decile 1 representing the most deprived and Decile 10 the least deprived. The data is available at the statistical sector level, with the most recent version based on the 2011 census. Due to the decennial nature of the census, these values remain the most up-to-date comprehensive measure.

In this study, the BIMD 2011 values were linked to two main air pollution metrics: annual mean concentrations of NO₂ and PM₂.₅, and the seasonal (summer-to-winter) ratio for these pollutants. A smoothed local regression approach (LOESS) was used to explore the relationships between deprivation and air pollution metrics. LOESS allows for flexible modelling of non-linear associations while maintaining interpretability^30^. Statistical computations and visualizations were performed using ggplot2^31^.

#### Case-Study: Mitigation potential of transport interventions to meet the WHO guidelines

For the regions of Flanders and Brussels, the Mobiscore is a recent tool developed by government instances that provide an overview of how well essential services such as education, groceries, doctors, recreational and sport services and railway stations are accessible within a reasonable distance (accessible by active or public transport) from place of residence. Plotting the Mobiscore against the number of private cars owned per household can give an indication of to what extent proximity of robust public transport and essential services can decrease car owns per household, knowing that, in many areas and especially cities in Europe motorised road transport is the primary cause of Nitrogen Dioxide exposure and one of the main sources, along a variety of sources including residential heating, industry and agriculture, of Particulate Matter^34–36^. Statistical computations and visualizations were performed using ggplot2^31^.

## Results

### Seasonal and spatial-seasonal patterns of PM_2.5_ and NO_2_

**Fig. 2:**
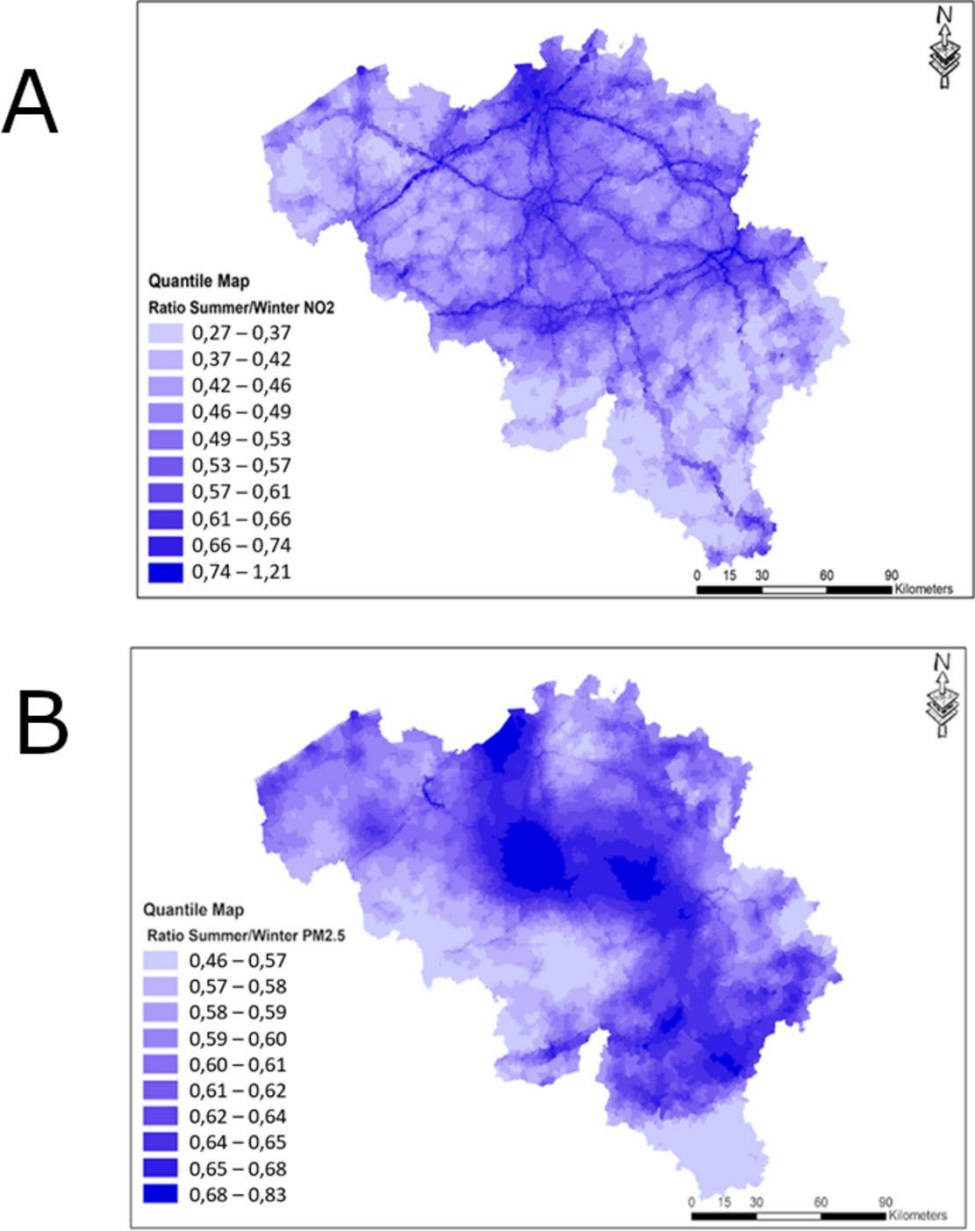
**(a)** Ratio NO_2_ concentrations summer/winter for the 2016 calendar year (quantile map with 10 deciles, mean value = 0.53). **(b)** Ratio PM_2.5_ concentrations summer/winter for the 2016 calendar year (quantile map with 10 deciles, mean value = 0.61).

NO_2_ concentrations close to the main traffic axis show the smallest difference between summer and winter (Fig. 2a). Further away from the traffic sources, NO_2_ pollution is more diluted and summer/winter differences are larger with summer values considerable lower compared to winter values. For PM_2.5_, there is a clear spatial variation in the summer/winter ratio, as a results of a variety of sources in combination with atmospheric processes (Fig. 2b).

**Fig. 3:**
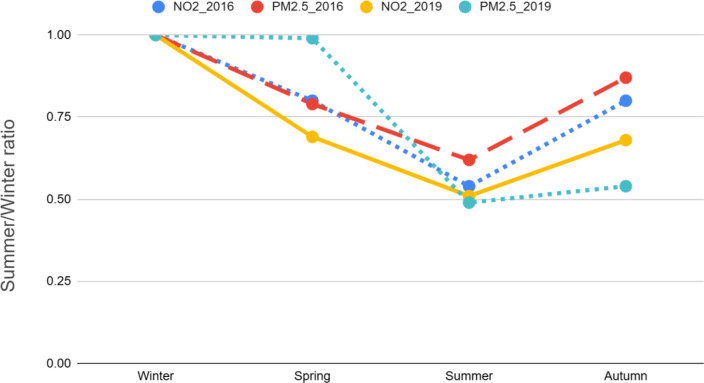
Relative NO_2_ and PM_2.5_ concentrations in Spring, Summer and Autumn compared to winter for the calendar years 2016 and 2019.

Figure 3 illustrates the seasonal pattern and shows average concentrations of NO_2_ and PM_2.5_ for the entirety of Belgium relative to the winter concentrations for the calendar years of 2016 and 2019. Both pollutants show clear reductions in summer of 35–55% compared to winter.

Stratifying by CORINE land cover type (Tables 1 and 2), it is clearly visible the summer/winter ratio close to 1.0 for NO_2_ occurs in the classes “road and rail networks and associated land” (0.78), water courses (0.75), port areas (0.74) and continuous urban fabric (0.69), while the concentrations are most diluted in peat bogs (0.41) and various types of forests such as broad-leaved forest (0.48), coniferous forest (0.48), mixed forest (0.48), traditional woodland-shrub (0.49) and natural grasslands (0.49).

For PM_2.5_, the variation in summer/winter ratio does differ to a much lesser extent amongst land cover class, with most land cover classes having a ratio that is close to each other (0.4–0.6), except for peat bogs with a 0.32 ratio.

Examining the degree of urbanisation (Table 3), NO_2_ summer/winter ratio is closer to 1 in urban compared to rural areas (0.57 vs. 0.46), which is also the case for PM_2.5_, but less outspoken (0.54 vs. 0.51).

**Fig. 4:**
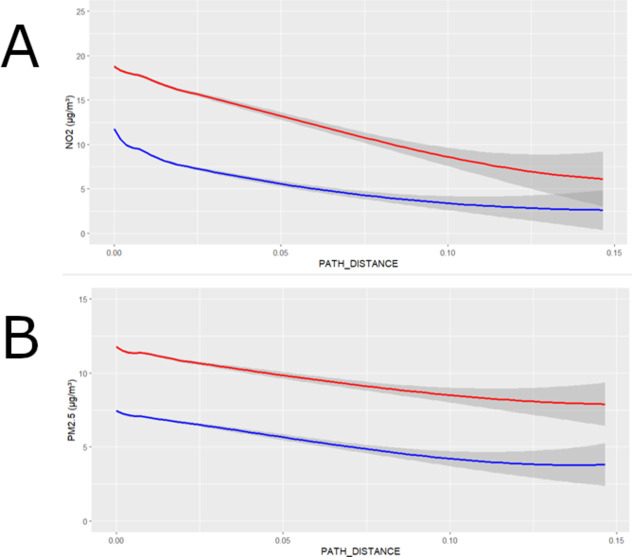
**(a)** The absolute values of NO_2_ in winter (red line) and summer (blue line) plotted against a function of the distance to motorways, primary and secondary roads. CI is shown. **(b)** The absolute values of PM_2.5_ in winter (red line) and summer (blue line) plotted against a function of the distance to motorways, primary and secondary roads. CI is shown.

As previously observed in the maps presented in Fig. 2, major roads can be easily recognised on the maps as having a summer/winter ratio closer to 1.0 compared to other parts of Belgium. Plotting the absolute values of NO_2_ and PM_2.5_ against the distance from motorways, primary and secondary roads, it is clear both PM_2.5_ and NO_2_ concentrations are higher close to motorways, primary and secondary roads and decrease by going further away from those landscape elements (Fig. 4-a and -b). When taking the path_distance = 0.05 as intersection, we see that as this intersection point NO_2_ in summer (blue line) decreased from 12.0 to 6.0 µg/m³ (−50%) from origin to intersection, while having decreased from 18.5 to 13.0 µg/m³ in winter from origin to the same intersection point (−30%), indicating a slower dilution of concentrations in winter away from the main source of traffic. For PM_2.5_ it goes from 12.0 to 9.9 µg/m³ in winter (−17,5%) and from 7.5 to 5.5 µg/m³ in summer (−27%), again indicating a faster dilution in summer. Also, PM_2.5_ dilutes slower compared to NO_2_ away from the traffic sources, which can be explained by the presence of multitude of sources for PM_2.5_.

**Table 1:** NO_2_ ratios for summer compared to winter as reference season, split by different land cover classes for the calendar year 2019. Absolute winter concentrations in µg/m³ are also indicated.

NO_2_ ratios, reference winter, landcover classes (calendar year 2019)	Winter Concentration [REF] µg/m³	Summer	%Area
Continuous urban fabric	28.4 +/- 5.8	0.69 [0.57–0.75]	0.2
Discontinuous urban fabric	18.2 +/- 4.9	0.56 [0.34–0.63]	16.9
Industrial or commercial units	21.2 +/- 5.2	0.63 [0.31–0.74]	1.8
**Road and rail networks and associated land**	26.3 +/- 7.3	**0.78** **[0.48–0.90]**	0.3
Port areas	28.9 +/- 4.5	0.74 [0.57–0.83]	0.2
Airports	19.0 +/- 4.8	0.56 [0.35–0.63]	0.2
Mineral extraction sites	15.0 +/- 3.6	0.54 [0.34–0.61]	0.2
Dump sites	23.2 +/- 6.0	0.66 [0.23–0.80]	0.0
Construction sites	19.9 +/- 6.2	0.63 [0.27–0.72]	0.1
Green urban areas	23.6 +/- 5.2	0.63 [0.33–0.75]	0.2
Sport and leisure facilities	17.4 +/- 5.1	0.54 [0.28–0.61]	0.7
Non-irrigated arable land	15.5 +/- 3.5	0.51 [0.32–0.58]	21.7
Fruit trees and berry plantations	17.8 +/- 2.2	0.53 [0.47–0.56]	0.3
Pastures	13.1 +/- 5.4	0.51 [0.11–0.56]	11.4
Complex cultivation patterns	15.6 +/- 5.2	0.52 [0.25–0.58]	17.3
Agriculture mixed with natural vegetation	15.9 +/- 5.3	0.53 [0.23–0.59]	6.1
Broad-leaved forest	11.1 +/- 4.8	0.48 [0.00–0.56]	6.8
Coniferous forest	10.4 +/- 5.4	0.48 [0.00–0.53]	4.4
Mixed forest	9.4 +/- 4.3	0.48 [0.00–0.55]	8.8
Natural grasslands	10.9 +/- 5.3	0.49 [0.00–0.58]	0.0
Moors and heathland	16.6 +/- 7.1	0.57 [0.00–0.69]	0.5
Transitional woodland-shrub	10.4 +/- 5.3	0.49 [0.00–0.55]	0.6
Beaches, dunes, sands	16.0 +/- 2.6	0.53 [0.38–0.60]	0.0
Inland marshes	16.8 +/- 5.2	0.53 [0.20–0.61]	0.1
Peat bogs	6.4 +/- 0.9	0.41 [0.38–0.43]	0.2
Salt marshes	20.1 +/- 5.5	0.63 [0.27–0.74]	0.0
Intertidal flats	16.4 +/- 3.3	0.55 [0.28–0.66]	0.1
Water courses	19.7 +/- 4.7	0.75 [0.53–0.83]	0.2
Water bodies	20.4 +/- 7.6	0.63 [0.00–0.82]	0.3
Estuaries	25.1 +/- 4.5	0.71 [0.43–0.84]	0.1
Sea and ocean	19.9 +/- 5.4	0.64 [0.10–0.80]	0.1

**Table 2:** PM_2.5_ ratios for the summer season compared to winter as reference season, split by different land cover classes for the calendar year 2019. Absolute winter concentrations (µg/m³) are also indicated.

PM_2.5_ ratios, reference winter, landcover classes (calendar year 2019)	Winter Concentration µg/m³ [REF]	Summer	% area
Continuous urban fabric	15.3 +/- 1.3	0.56 [0.47–0.62]	0.2
Discontinuous urban fabric	14.2 +/- 2.2	0.48 [0.29–0.58]	16.9
Industrial or commercial units	15.2 +/- 1.8	0.51 [0.36–0.60]	1.8
Road and rail networks and associated land	15.2 +/- 2.0	0.56 [0.33–0.62]	0.3
Port areas	16.4 +/- 1.4	0.57 [0.55–0.59]	0.2
Airports	14.2 +/- 1.9	0.49 [0.34–0.58]	0.2
Mineral extraction sites	13.5 +/- 1.8	0.42 [0.28–0.50]	0.2
Dump sites	16.6 +/- 1.7	0.50 [0.34–0.60]	0.0
Construction sites	14.4 +/- 2.2	0.50 [0.33–0.60]	0.1
Green urban areas	14.2 +/- 1.5	0.53 [0.41–0.61]	0.2
Sport and leisure facilities	13.9 +/- 2.3	0.49 [0.28–0.59]	0.7
Non-irrigated arable land	13.3 +/- 1.8	0.46 [0.26–0.57]	21.7
Fruit trees and berry plantations	15.6 +/- 1.6	0.46 [0.39–0.51]	0.3
Pastures	12.4 +/- 2.9	0.45 [0.13–0.57]	11.4
Complex cultivation patterns	13.6 +/- 2.7	0.49 [0.25–0.59]	17.3
Agriculture mixed with natural vegetation	13.7 +/- 2.7	0.47 [0.25–0.57]	6.1
Broad-leaved forest	11.2 +/- 2.2	0.40 [0.18–0.50]	6.8
Coniferous forest	11.4 +/- 3.3	0.42 [0.04–0.54]	4.4
Mixed forest	10.5 +/- 2.2	0.40 [0.16–0.49]	8.8
Natural grasslands	11.0 +/- 1.9	0.43 [0.08–0.61]	0.0
Moors and heathland	14.5 +/- 2.9	0.49 [0.27–0.59	0.5
Transitional woodland-shrub	11.3 +/- 3.2	0.42 [0.05–0.52]	0.6
Beaches, dunes, sands	13.2 +/- 2.0	0.55 [0.54–0.57]	0.0
Inland marshes	14.3 +/- 2.5	0.49 [0.32–0.58]	0.1
Peat bogs	9.3 +/- 0.9	0.32 [0.24–0.37]	0.2
Salt marshes	14.0 +/- 1.8	0.55 [0.52–0.57]	0.0
Intertidal flats	12.2 +/- 1.0	0.56 [0.54–0.57]	0.1
Water courses	14.6 +/- 2.0	0.48 [0.26–0.60]	0.2
Water bodies	15.3 +/- 2.3	0.51 [0.34–0.61]	0.3
Estuaries	15.7 +/- 1.0	0.57 [0.55–0.58]	0.1
Sea and ocean	12.7 +/- 1.2	0.56 [0.52–0.60]	0.1

**Table 3:** Ratio of NO_2_ and PM_2.5_ in summer compared to winter for the calendar year 2019.

Degree of Urbanisation	Ratio NO_2_ summer/winter	Ratio PM_2.5_ Summer/Winter
1 City	0.57	0.54
2 Towns and Suburbs	0.51	0.54
3 Rural Areas	0.46	0.51

### Seasonal contributions to annual air pollution and annual preventable fraction

**Table 4:** Contributions of winter, spring, summer and autumn concentrations to annual residential exposure. The last column displays the annual preventable fraction (PF) including CI’s for the counterfactual scenario of reaching the WHO guidelines of 5 µg/m³ PM_2.5_ and 10 µg/m³ NO_2_ long-term exposure.

%Contribution to annual residential exposure	Winter	Spring	Summer	Autumn	Annual PF
NO_2_	43.9%	23.6%	9.8%	22.8%	3.1% [2.0–4.6%]
PM_2.5_	38.3%	37.5%	10.9%	13.3%	6.4% [3.4–9.3%]

The seasonal contributions to annual residential exposure to air pollution, as presented in Table 4, reveal distinct seasonal variations in the exposure levels for NO₂ and PM₂.₅. Winter emerges as the season with the highest contribution, accounting for 43.9% of the annual residential exposure to NO₂ and 38.3% for PM₂.₅. In contrast, summer contributes the least, with only 9.8% and 10.9% of annual exposure for NO₂ and PM₂.₅, respectively. Spring and autumn demonstrate intermediate levels of contribution, with spring contributing 23.6% for NO₂ and 37.5% for PM₂.₅, while autumn contributes 22.8% for NO₂ and 13.3% for PM₂.₅.

#### Socio-economic disparities

**Fig. 5:**
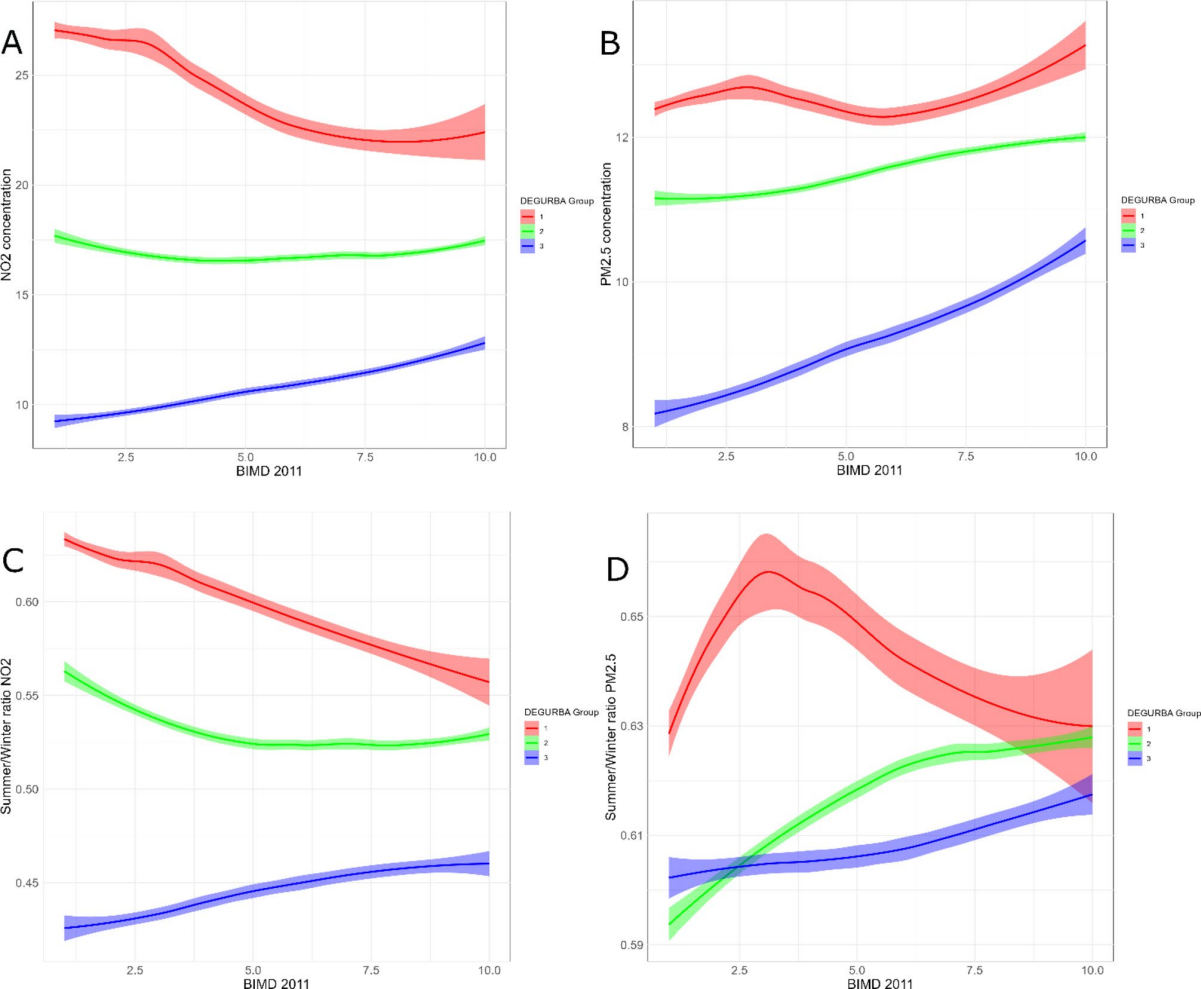
(**a**) NO2 concentrations plotted against the BIMD 2011 index. A lower BIMD index value implies a higher level of deprivation (1= most deprived, 10 = least deprived) (**b**) PM2.5 concentrations plotted against the BIMD 2011 index. A lower BIMD index value implies a higher level of deprivation (1= most deprived, 10 = least deprived). (**c**) NO_2_ summer/winter ratio plotted against the BIMD 2011 Index. A lower BIMD index value implies more deprivation (1= most deprived, 10 = least deprived) (**d**) PM2.5 summer/winter ratio plotted against the BIMD 2011 Index. A lower BIMD index value implies more deprivation (1= most deprived, 10 = least deprived)

For NO₂ (Fig. 5-a), high nitrogen dioxide levels in urban areas (DEGURBA = 1) are strongly associated with areas of highest deprivation. The most deprived people in urban areas are clearly exposed to the highest NO₂ concentrations, while those with moderate or low levels of deprivation experiencing lower exposure. In suburban areas and towns (DEGURBA = 2), NO₂ concentrations remain relatively constant across all deprivation levels. In contrast, rural areas (DEGURBA = 3) show an opposite trend to urban areas: NO₂ concentrations are higher among the least deprived groups. Overall, NO₂ concentrations are highest in urban areas and lowest in rural areas.

The relationship between deprivation and PM₂.₅ differs (Fig. 5-b). Although the variations are small, in urban areas, the least deprived individuals tend to be exposed to slightly higher PM₂.₅ levels compared to the most deprived groups. This trend is more pronounced in rural areas (DEGURBA = 3), where a clear gradient emerges: highly deprived individuals are exposed to the lowest PM₂.₅ concentrations, while the least deprived experience the highest concentrations.

Figures 5-c and 5-d illustrate the summer-to-winter ratio for NO₂ and PM₂.₅. For NO₂, the summer/winter ratio is highest in the most deprived areas (lower deciles), with values around 0.65, compared to 0.55 in urban areas where the least deprived people generally live. In suburban areas and towns (DEGURBA = 2), this relationship persists but is less pronounced. In rural areas, the trend reverses, with slightly higher summer/winter ratios observed among the least deprived groups. Overall, the summer/winter ratios for NO₂ are highest in urban areas and lowest in rural regions.

For PM₂.₅, the pattern is different. In urban areas, the summer/winter ratio initially increases with decreasing deprivation, peaking among individuals in the 3rd deprivation decile (where 70% of the population is less deprived, and 20% is more deprived). Beyond the 3rd decile, the summer/winter ratio decreases as deprivation decreases further. In suburban and rural areas, however, the summer/winter ratio increases steadily with decreasing deprivation (Fig. 5-d).

#### Case-study: Exploring the Association Between Mobiscore and cars owned per household and implications to reach WHO guidelines for PM_2.5_ and NO_2_

**Fig. 6:**
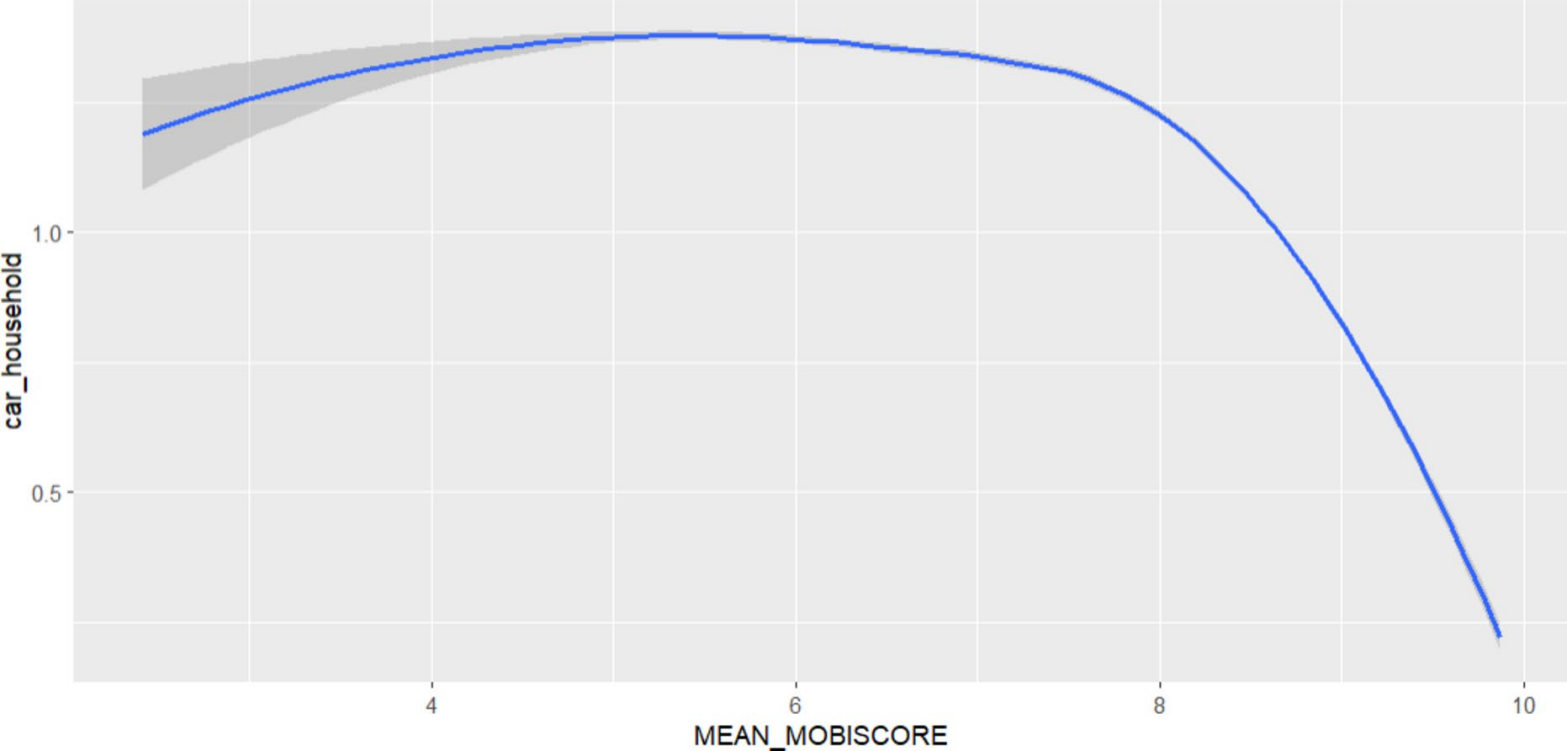
Localised regressions plotted for cars owned per household versus mean Mobiscore per statistical sector.

In terms of the mitigation potential through transport & spatial planning interventions, we look to the number of cars owned per household plotted against the Mobiscore. On the localised regression (Fig. 6), it is visible that from a cut-off score around 8.0 for the Mobiscore, a rapid decrease of the number of cars owned per household is initiated.

## Discussion

### Discussion and interpretation of research results

NO₂ and PM₂.₅ show notable seasonal variations, with summer concentrations being 35–50% lower for PM₂.₅ and over 45% lower for NO₂ compared to winter levels. These seasonal differences can be attributed to both changes in emissions and variations in atmospheric conditions. During winter, increased emissions from residential heating, energy production, and transportation, particularly in urban areas, contribute to higher PM_2.5_levels^37,38^. Additionally, atmospheric conditions such as temperature inversions, reduced solar radiation, and lower wind speeds hinder the dispersion of pollutants, leading to higher winter concentrations. In contrast, summer sees lower emissions from heating and more favourable meteorological conditions, including stronger sunlight, higher temperatures, and increased atmospheric mixing, which enhance the dispersion and chemical transformation of pollutants^39,40^.

Our analysis also found that these seasonal changes vary by location, highlighting an important spatial pattern for both pollutants. It is important to acknowledge that the ratio between summer and winter concentrations near highly trafficked roads and densely populated urban areas tends to be higher, and even around 1.0 near the busiest roads, such as motorways, for NO_2_. This indicates limited pollution dilution in summer for individuals residing in these locations close to traffic hotspots, who therefore do not benefit from the enhanced pollution dilution in summer. Conversely, those living further away from busy roads experience more significant summer vs. winter dilution of air pollution, with autumn and spring being characterised by intermediate values. A stratification of NO_2_ ratios by land cover classes unravels a very clear pattern between the ratios and land cover classes with ports, roads and urban centres having a summer/winter ratio most close to 1.0.

For PM_2.5_, there are also clear spatial components of the seasonal patterns, with in general major urban centres having a ratio closer to 1.0, although patterns are less straightforward to interpret as for NO_2_, and a comparison of the ratio amongst land cover classes shows much weaker distinctions in summer/winter ratio as is the case for NO_2_. As for NO_2_, PM_2.5_ values also tend to decrease further away from main traffic axes.

The observed spatial patterns in summer-to-winter ratios for NO_2_ and PM_2.5_ can be attributed to a combination of factors, including traffic emissions, urban heat island effects, and local meteorology. Near heavily trafficked roads, higher NO_2_ratios in summer, sometimes approaching 1.0, likely result from persistent emissions coupled with very limited vertical mixing due to low wind speeds in dense urban environments^9^. The lack of significant reduction in summer concentrations near motorways and traffic hotspots indicates that these locations experience continuous emissions from vehicles. Furthermore, the urban heat island effect can induce circulation patterns, such as the urban breeze, which can exacerbate pollution retention in city centres by altering local wind fields and creating areas of convergent flow^41^. For PM_2.5_, while a less distinct summer-to-winter spatial pattern is evident, the weaker differentiation across land cover classes could stem from the more diffuse nature of PM_2.5_sources, including secondary aerosol formation and contributions from natural sources or agricultural activities^42^. The weaker seasonal distinctions in PM_2.5_ concentrations also highlight the role of long-range transport, which can override local source contributions These findings underscore the challenge of achieving long-term WHO air pollution guidelines, particularly in urban areas and traffic hotspots where persistent emissions and limited seasonal dilution maintain high pollutant concentrations year-round. Effective mitigation strategies must prioritise reducing emissions at the source, especially in densely populated urban centres and along major traffic axes, to ensure progress toward meeting WHO guideline values for NO₂ and PM₂.₅.

The observed relationships between deprivation, DEGURBA classification, and air pollutant levels highlight significant disparities in exposure to NO₂ and PM₂.₅, which directly impact efforts to achieve the WHO long-term air quality targets of 10 µg/m³ for NO₂ and 5 µg/m³ for PM₂.₅. In urban areas, the strong association between high NO₂ concentrations and the most deprived populations reflects the burden of traffic-related emissions in densely populated, socioeconomically disadvantaged areas. These groups often have limited capacity to mitigate exposure, underscoring the need for targeted policies. As example, a study in the United Kingdom demonstrated that congestion charging had a strong potential in reducing NO_2 _and the related health burden, with a stronger effect (larger improvement) for socioeconomically disadvantaged areas^43^.

The weaker relationship between PM₂.₅ and deprivation can be attributed to its complex nature as a mixture of pollutants and the significant influence of long-range transport, which often overrides local socioeconomic and emission patterns. A previous study in the Netherlands and the UK found stronger associations between NO₂ and deprivation compared to PM. However, variations between countries and regions were noted, and significant links between higher PM₁₀ levels and increased deprivation were often observed^44^. This contrasts with the inverse relationship, particularly in rural areas, identified in our study.

The Mobiscore defines to what degree reliable public transport is present at residential addresses, and takes into account how well education facilities, grocery shopping, recreational activities, railway stations and healthcare services are reachable by active or public transport. An earlier study concluded that proximity of services reduces the frequency of car-use, but they do not alter travel distances for people continuing to use cars^45^. This implies that car ownership is a valuable indicator for assessing the effectiveness of service proximity and 15-minute communities, as the absence of a car strongly suggests a shift away from car usage. Our analysis demonstrates that starting from a Mobiscore of 8.0, a sharp decrease in number of cars owned per households is initiated, potentially signalling that increasing the Mobiscore of certain areas could result in decreased car ownership per household, which would in turn result in lower NO_2_ and to a lesser extent lower PM_2.5_ exposure.

### Strengths and limitations

Our study has notable strengths. Firstly, we utilise the most up-to-date and comprehensive Exposure Response Functions (ERFs) available, reflecting the increasingly established link between air pollution and public health. Recent ERF functions, like those from the ELAPSE meta-analysis, incorporate the latest and largest studies to date investigating the health effects of low-level air pollution. Including more recent studies is advantageous as data for such studies continuously improves with more accurate exposure assessment techniques. There has also been a sharp increase in recent years in the number of studies investigating the relationship between air pollution and mortality. The ELAPSE meta-analysis is the first to include a considerable amount of studies on NO_2_ that cover areas with low levels of air pollution. Additionally, while conducting an Health Impact Assessment in Belgium, focusing on the European region is important due to the significant variability in air pollutant composition, particularly PM_2.5_, across different parts of the world. Research demonstrated that traffic-related and biomass burning PM_2.5_ have stronger health effects compared to industrial or soil-derived PM_2.5_^46,47^.This variation is attributed to differences in chemical composition, particularly the presence of Black Carbon (BC) and Ultrafine Particles (UFP), which are known to cause severe health effects. Another strength is that the ELAPSE ERFs used from the meta-analysis are all corrected for socio-economic factors and confounding factors such as smoking. Secondly, our study benefits from high-resolution air pollution data, which are aggregated to the statistical sector level, enabling the identification of spatial patterns at various geographical levels. We also consider the spatial component of seasonal patterns. This has important implications for policy making, as for example for both PM_2.5_ and NO_2_ only ca. 10% of residential exposure occurs in the summer season, while very heavily trafficked locations have high exposure all-year round with very small seasonal differences. Understanding these patterns is important to be able to identify the right mitigation measures to reduce the air pollution concentrations below the WHO guidelines of 5 µg/m³ PM_2.5_ and 10 µg/m³ NO_2_ exposure. Therefore, our approach offers valuable information and data interpretation for public health and environmental policymakers.

In our study, we acknowledge certain limitations. A limitation of this study is the exclusion of ozone (O_3_), which, despite its distinct spatio-temporal dynamics compared to NO_2_ and PM_2.5_, also poses significant health risks. Within our study area, O_3_ is primarily relevant during the summer season, whereas NO_2_ and PM_2.5_ contribute to health risks across all seasons despite seasonal concentration variations. By focusing on pollutants with year-round relevance, this study provides actionable insights for urban air quality management. However, the omission of O_3_ may reduce the study’s comprehensiveness, particularly in capturing summer-specific health impacts. Future research could benefit from integrating O_3_ to complement the findings and provide a more holistic understanding of air pollution’s health burden.

One other notable limitation is the restricted temporal scope of our data, which is confined to the years 2016 and 2019. Including additional years could enhance the robustness of the analysis, but it also introduces a considerable trade-off due to the substantial computational time required for high-resolution models.

Another issue is the amalgamation of road and rail networks into a single land cover class in the CORINE land cover classification. Our research, supported by existing literature, suggests that road networks might exhibit distinct characteristics, such as higher absolute concentrations and a closer summer/winter ratio, compared to rail networks. This distinction could not be adequately addressed due to the combined classification. A potential solution for this is using additional datasets containing primary and secondary roads, to identify relations in this subset, omitting rail networks from this class. Further, a limitation is that we deal with residential exposure only for calculating Preventable Fractions (PFs). Both the ERFs used from literature as well the air pollution data used in our study, are based on residential exposure. While this is true for most or nearly all existing health impact assessment studies, it would be better if all-day exposure data are used, including residential indoor exposure, both in the epidemiological cohort studies to identify ERFS and subsequently in the Health Impact Assessment exercise. Technological advancements in air pollution measurements, such as mobile monitoring devices that also could track indoor exposure, or coupling of smartphone based location data with air pollution maps, feed the hope that such analysis will be possible in the future^47–49^. Also, the seasonal analysis assumes that the duration of residential exposure remains consistent across seasons, which is a key consideration when interpreting seasonal contributions to annual exposure. Variations in time spent indoors or outdoors across seasons, and other behavioural factors, could influence the actual exposure levels and should be explored in future studies to refine these estimates further.

Another limitation is, especially for PM_2.5_, that we could not differentiate explicitly between local and regional emissions and long-range emissions causing air pollution exposure. Long-range transported PM_2.5_ pollution plays a substantial role in Belgium’s air quality, with 40–55% of PM_2.5_ exposure originating domestically, according to the Urban PM_2.5_Atlas^50^. For example, in Antwerp, 35–40% of PM_2.5_is sourced locally, about 20% from the rest of Belgium, and 40–45% from transboundary pollution^50^. These contributions are shaped by atmospheric processes and emission patterns in neighbouring countries, which often resemble those in Belgium. This context highlights the relevance of our spatial-seasonal analysis, even without explicitly differentiating between local and long-range emissions.

The case study in the third part of our research focuses specifically on traffic-related air pollution and mitigation strategies, such as analysing the association between mobiscore (takes into account number of services and public transport available) and reduced car ownership, rather than offering a comprehensive approach to reducing overall air pollution. This targeted approach underscores the importance of addressing local and regional emissions from transportation within the broader context of air quality management.

#### Implications for research and policy

Our study underscores the critical need for a deep comprehension of both seasonal and spatio-seasonal patterns. A thorough understanding of these patterns is essential to practically achieve the World Health Organization’s target levels of 5 µg/m³ for PM_2.5_ and 10 µg/m³ for NO_2 _for long-term exposure. For instance, seasonally targeted measures, can alleviate pressure on hospitals by lowering the occurrence and severity of cardiovascular events, cerebral events, and respiratory tract infections. By ensuring adequate hospital capacity during peak viral infection seasons like influenza, RSV, and COVID-19, interventions taking into account the presence of spatio-seasonal concentration differences can effectively contribute in managing the seasonal inequality in healthcare demand^51–53^.

Trafficked areas demonstrate elevated air pollution concentrations all-year round with minimal seasonal disparities. We also demonstrate that efforts to increase public transport availability and increase proximity of healthcare, education and recreational activities, and accessibility of those services with public and active transport, which results in an increased Mobiscore, starting from a certain threshold of services available, may effectively reduce car ownership per household, resulting in reduced NO_2_ and to a lesser extent reduced PM_2.5_ concentrations. This is an important aspect as our analysis revealed, especially for NO_2_, absolute concentrations are the highest close to busy roads. In addition, close to many busy roads such as motorways and urban traffic axes, the ratio summer/winter concentration is very close to 1.0, indicating reaching the WHO guidelines across Belgium is impossible without tackling those traffic-related hotspots.

Socioeconomic disparities play an important role in shaping exposure to air pollution, as our findings reveal higher NO₂ concentrations in urban areas where the most deprived populations reside. This highlights the need for targeted policies addressing air quality in these disadvantaged areas to reduce health inequities. Simultaneously, the inverse pattern for PM₂.₅ exposure in rural areas emphasizes the importance of considering socioeconomic factors alongside emission sources and long-range transport in designing mitigation strategies.

## Conclusion

In conclusion, our study underscores the significant spatial-seasonal patterns in air pollution concentrations and their implications for human health. In order to achieve the WHO guidelines of 5 µg/m³ PM_2.5_ and 10 µg/m³ NO_2_ long-term exposure, special attention should be dedicated to the winter season and trafficked areas with all-year round strongly elevated air pollution concentrations. On one hand, achieving WHO guidelines is impossible without season-specific measures while urban traffic hotspots show minimal seasonal reductions, emphasizing the need for structural, year-round mitigation measures.

Socioeconomic disparities further highlight the unequal distribution of exposure, with vulnerable populations often residing in areas with persistently higher NO_2_ pollution levels. Policies that prioritise reducing these disparities are essential, as they address both environmental justice and public health objectives.

In addition, our findings emphasize the potential of targeted urban planning strategies, such as improving public transport availability and promoting 15-minute communities, to mitigate traffic-related air pollution. There are strong associations between these measures and reduced car dependency, indicating these measures may reduce car dependency and lower NO₂ and PM₂.₅ emissions, altough a causal effect cannot be proven based on our analysis.

## Data Availability

- Seasonal air pollution data maps: those are extracted (postprocessed) from the high-resolution ATMOSTREET air pollution model and are available for free upon request (info@irceline.be) - Number of cars owned per household: open data dataset from STATBEL, available to download via the STATBEL website: https://statbel.fgov.be/en/themes/mobility/traffic/vehicles-household#:~:text=In%202022%2C%20Belgian%20households%20had%20on%20average%201.06%20cars%2C%20as,nature%20of%20the%20Brussels%20territory. - Degree of urbanisation < EUROSTAT/EEA: data are available on https://www.eea.europa.eu/data-and-maps/data/external/degree-of-urbanisation-degurba - MOBISCORE: data originating from Flemish Government. Data are avaialble to download via: https://www.vlaanderen.be/datavindplaats/catalogus/mobiscore-per-ha - Landcover: derived from CORINE 2018 landcover < Copernicus land monitoring service, data are available to download via https://land.copernicus.eu/en/products/corine-land-cover - Belgian Index of Multiple Deprivation BIMD 2011: Visualisation via https://bimd.sciensano.be/tool, download possible via Github: https://github.com/bimd-project/bimd.
